# Accelerated Electron Ionization-Induced Changes in the Myenteric Plexus of the Rat Stomach

**DOI:** 10.3390/ijms25126807

**Published:** 2024-06-20

**Authors:** Raina Ardasheva, Veselin Popov, Viktor Yotov, Natalia Prissadova, Mina Pencheva, Iva Slavova, Valentin Turiyski, Athanas Krastev

**Affiliations:** 1 Department of Medical Physics and Biophysics, Faculty of Pharmacy, Medical University of Plovdiv, 4002 Plovdiv, Bulgaria; rayna.ardasheva@mu-plovdiv.bg (R.A.); nataliya.prisadova@mu-plovdiv.bg (N.P.); mina.pencheva@mu-plovdiv.bg (M.P.); valentin.turiyski@mu-plovdiv.bg (V.T.); 2 Section of Radiotherapy and Nuclear Medicine, Department of Clinical Oncology, Faculty of Medicine, Medical University of Plovdiv, 4002 Plovdiv, Bulgaria; veselin.popov@mu-plovdiv.bg; 3 Department of Chemical Sciences, Faculty of Pharmacy, Medical University of Plovdiv, 4002 Plovdiv, Bulgaria; iva.slavova@mu-plovdiv.bg; 4 Medical College, Trakia University, 6015 Stara Zagora, Bulgaria; athanas_kristev@yahoo.com

**Keywords:** irradiation with accelerated electrons, gastric smooth muscles, enteric nervous system, electrical stimulation, myenteric plexus

## Abstract

The influence of accelerated electrons on neuronal structures is scarcely explored compared to gamma and X-rays. This study aims to investigate the effects of accelerated electron radiation on some pivotal neurotransmitter circuits (cholinergic and serotonergic) of rats’ myenteric plexus. Male Wistar rats were irradiated with an electron beam (9 MeV, 5 Gy) generated by a multimodality linear accelerator. The contractile activity of isolated smooth muscle samples from the gastric corpus was measured. Furthermore, an electrical stimulation (200 μs, 20 Hz, 50 s, 60 V) was performed on the samples and an assessment of the cholinergic and serotonergic circuits was made. Five days after irradiation, the recorded mechanical responses were biphasic—contraction/relaxation in controls and contraction/contraction in irradiated samples. The nature of the contractile phase of control samples was cholinergic with serotonin involvement. The relaxation phase involved ACh-induced nitric oxide release from gastric neurons. There was a significant increase in serotonergic involvement during the first and second contractile phases of the irradiated samples, along with a diminished role of acetylcholine in the first phase. This study demonstrates an increased involvement of serotonergic neurotransmitter circuits in the gastric myenteric plexus caused by radiation with accelerated electrons.

## 1. Introduction

It is well known that ionizing radiation is a factor destructively influencing the structure and functions of tissues [1]. It can disrupt various cellular and extracellular structures, affecting their pivotal constituent biomolecules—proteins, lipids, and nucleic acids [2], either by direct supply of energy to these macromolecules or by generating reactive oxygen species [3,4]. The degree of damage depends on the type of radiation, the absorbed dose, and the type of irradiated tissues, depending on their tissue weight coefficient [5].

Electron radiation has specific physical mechanisms of penetration and interaction with matter, different from those of electromagnetic ionizing radiation. Depending on their energy, flows of accelerated electrons penetrate different depths in tissues and phantom models [6,7,8]. In this sense, they provoke specific ionization-induced effects in targeted tissues, leading to disturbances in their cellular functions [9], membrane permeability, enzyme reactions [10], etc.

Disruptions of neurotransmitter circuits affect all processes in the body and can cause an imbalance in the functions of target tissues, organs, and systems. It was generally assumed that nervous tissue was relatively resistant to radiation and that only high doses could cause some damage because there is no active cell division in normal mature nervous tissue [11]. However, there is evidence that the nervous system responds to even small doses of radiation. Recent studies show that despite neuronal cell loss due to apoptosis in the myenteric ganglia of adult individuals, the total number of myenteric neurons remains relatively constant [12]. This so-called neuronal homeostasis is maintained by newly formed neurons in vivo from dividing precursor cells localized in these ionizing radiation-sensitive ganglia [13]. Another study by Kempf et al. demonstrates that ionizing radiation immediately impairs synaptic plasticity associated with molecular signaling pathways in the mouse hippocampus and cerebral cortex [14].

It is well known that neurotransmitters are essential in carrying out neuronal functions. Many neuronal dysfunctions after radiation exposure result from damage to structures involved in various steps of the transmission process: synthesis and secretion of neurotransmitters, expression and development of transport cell structures, properly functioning enzyme systems, receptors, etc. [15,16]. Quantities of some neurotransmitters, namely serotonin, dopamine, and GABA, are significantly reduced in brain tissue and the hippocampus in mice after X-ray whole-body irradiation [12].

To date, whether and to what extent accelerated electrons cause changes to the functional capacity of neurotransmitter circuits has not been unequivocally determined. The synthesis and action of most neurotransmitters take place in the central nervous system (CNS) and enteric nervous system (ENS). Studies discussing the effects of ionizing radiation are mainly focused on CNS neurons [12,17]. At the same time, the diversity of neurons in the ENS, which communicate with each other and with other cell types through a variety of neurotransmitters, neuromodulators, and peptides [18], makes it a suitable object for studying the effect of ionizing radiation on multiple signaling pathways.

Some early reports have found an increase in the activity of dopaminergic and cholinergic systems in the rat brain after high-energy electron irradiation [19]. However, there is an insufficient number of studies concerning the effects of accelerated electrons on the ENS and particularly the myenteric plexus, as part of it. In this study, we aimed to clarify the influence of accelerated electron fluxes on some pivotal neurotransmitter circuits of the rat myenteric plexus. For this purpose, we used gastric smooth muscle (SM) samples as a model for testing them (see Section 4.1).

## 2. Results

### 2.1. Characteristics of Spontaneous Contractile Activity of SM Samples from Control and Irradiated Rats

Five days after the irradiation procedure, the results indicated no alterations in spontaneous biomechanical activity. There were no measurable differences in the frequency and amplitude of spontaneous contractions of SM samples from control and irradiated rats (Table 1).

### 2.2. Electrically Induced SM Responses

**Figure 1:**
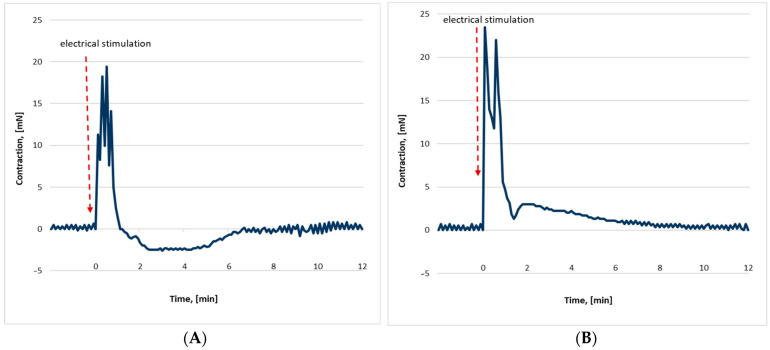
Representative records of electrically elicited SM mechanical responses of control (**A**) and irradiated (**B**) rat samples.

The mechanical SM reactions (n = 9) of the control samples provoked by electrostimulation were biphasic—contractile/relaxation (Figure 1A). The contractile phase had a maximum force of 19.38 ± 3.23 mN and a duration of 53.0 ± 7.0 s. Its subsequent relaxation phase reached a maximum value of −1.87 ± 0.64 mN and a duration of 225.9 ± 92.7 s until complete recovery of the initial tone.

SM samples (n = 7) from the irradiated rats responded to electrical stimulation with a biphasic response of two distinguishable sequential contractions (Figure 1B). The first contractile phase reached a maximum force of 21.87 ± 7.16 mN and a duration of 49.8 ± 5.3 s, and the second 2.35 ± 0.83 mN and 238.8 ± 85.8 s.

A comparison of both phases between the groups was performed. It showed a non-significant difference in the first phase and a significant one in the second phase (a change in the type of reaction to the stimuli).

To understand the nature of electrically induced SM contractions in all samples, we conducted experiments with different blockers, antagonists, and agonists.

### 2.3. Effect of Tetrodotoxin (TTX) on Electrically Induced Mechanical Responses

It is well known that TTX is a voltage-gated sodium channel blocker. In both groups of samples, TTX (5 × 10^−7^ mol/L) prevented any SM responses caused by electrical stimulation with the parameters indicated above (n = 4). These results indicate neuronal involvement in the reactions.

### 2.4. Effect of Electron Radiation on Elements of Cholinergic Transmission

To assess the involvement of the cholinergic system, atropine (1 × 10^−6^ mol/L) and 4-DAMP (2 × 10^−6^ mol/L), both m-cholinoreceptors blockers, were used. Furthermore, a quantile measurement of acetylcholine esterase (AChE) in SM homogenates from both groups was performed.

#### 2.4.1. Experiments with Muscarinic Receptor Antagonists

The blockers had a different strength and nature of influence on the biphasic electrically induced SM responses in control and irradiated samples, as visible from the results presented in Table 2. 4-DAMP tendentiously reduced the force of SM contraction in the first phase from both groups compared to their initial values. Also, the blocker tendentiously reduced the amplitude of the second phase in the control samples compared to their initial value, while in the irradiated samples, no observable alterations were found in the same phase.

The electrically induced first phase of the reaction after the application of atropine was tendentiously reduced in both groups compared to their initial values. Interesting results were observed in the second phase of the control samples after applying atropine. It tended to block the relaxation phase in them (Table 2). Atropine tendentiously increased the amplitude of the second phase in the irradiated samples compared to their initial value.

#### 2.4.2. AChE Activity

**Figure 2:**
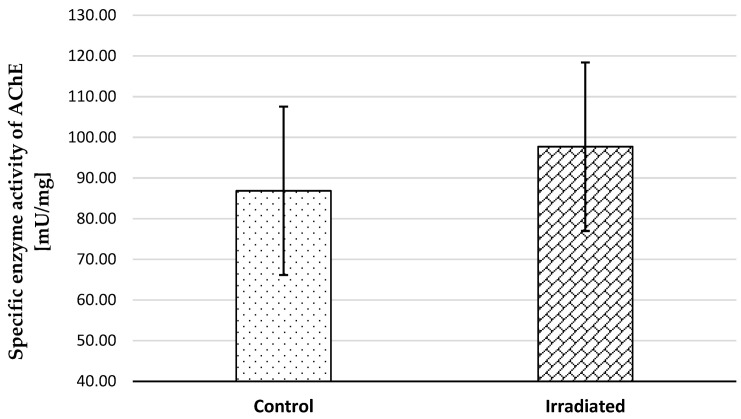
Quantitate measurement of AChE activity in gastric SM tissue homogenates obtained from control (n = 11) and irradiated (n = 11) samples.

The AChE activity of control and irradiated samples was investigated and compared in tissue homogenates from both groups. There was a tendency towards an increased activity of the enzyme in the irradiated samples compared to the control (Figure 2).

### 2.5. Influence of Electronic Radiation on Elements of Serotonergic Transmission

To assess the involvement of the serotonergic system, ketanserin in the concentration of 1 × 10^−6^ mol/L (a selective 5-HT_2_ receptor antagonist) was used. Furthermore, a quantile measurement of 5-HT_2A_ and 5-HT_2B_ receptors population in the myenteric plexus was performed by immunohistochemical studies.

#### 2.5.1. Functional Experiments on 5-HT_2A_ and 5-HT_2B_ Receptors

The effects of electron radiation on serotonergic transmission were functionally studied by comparing the influence of ketanserin on the electrically evoked responses of control and irradiated samples (Table 3). The first phase of contraction in the control samples was not affected by the application of the blocker compared to their initial value. However, ketanserin statistically reduced the first phase of the irradiated samples compared to their initial value. A statistical decrease in the contraction (first phase) from the irradiated samples was observable compared to the control ones.

The relaxation phase of the control samples was statistically increased (in absolute value) upon application of the 5-HT_2_-blocker compared to its initial value. Furthermore, ketanserin statistically reduced the second phase of the irradiated samples compared to their initial value.

#### 2.5.2. Light-Microscopic Immunohistochemical Analysis of 5-HT_2A_ and 5-HT_2B_ Receptors

**Figure 3:**
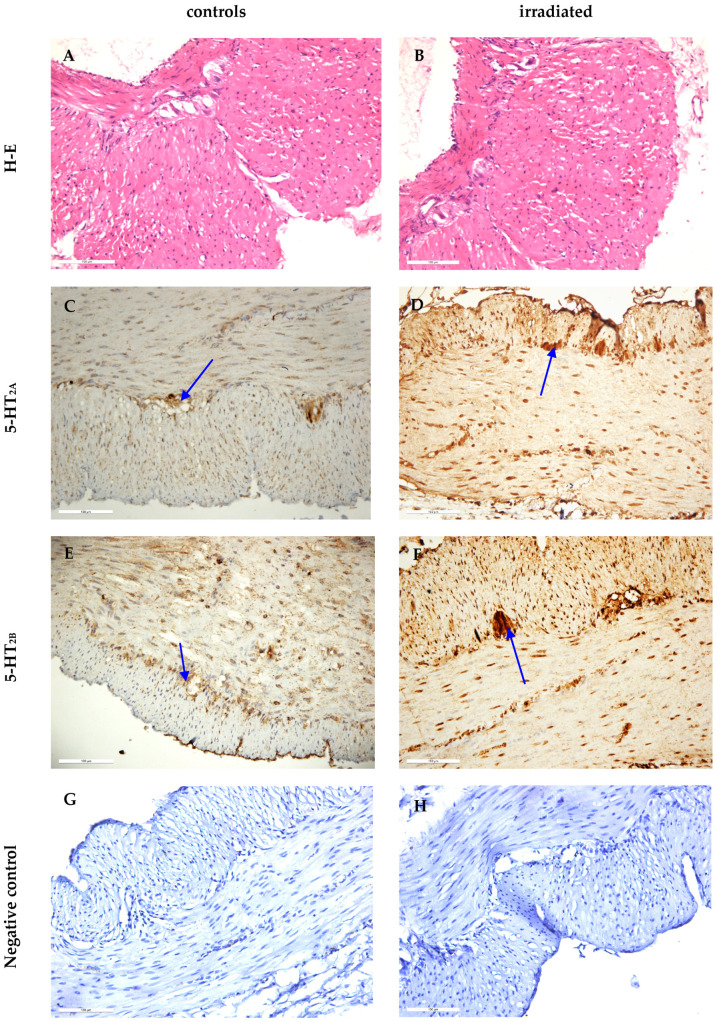
(**A**) Control samples stained with H-E; (**B**) irradiated samples stained with H-E; immunohistochemical reactions of the: (**C**) 5-HT_2A_ receptor in control samples; (**D**) 5-HT_2A_ receptor in irradiated samples; (**E**) 5-HT_2B_ receptor in control samples; (**F**) 5-HT_2B_ receptor in irradiated samples. In all images, the immunohistochemical reactions of both receptors in myenteric plexus neurons are marked with blue arrows. (**G**) Negative controls of the 5-HT_2A_ receptor by the omitting of primary antibodies in the tissue sample. (**H**) Negative controls of the 5-HT_2B_ receptor by the omitting of primary antibodies in the tissue sample. The magnification for all images is ×200.

Light-microscopic immunohistochemical reactions of 5-HT_2A_ and 5-HT_2B_ receptors were determined in myenteric plexus neurons of samples from control and irradiated rats (Figure 3). There were no visible morphological changes between hematoxylin and eosin (H-E) stained samples of the two groups (A and B). We found an increased immunohistochemical reaction of the 5-HT_2A_ receptor from irradiated animals compared to controls (C and D). Regarding the 5-HT_2B_ receptor, a similar increase of the reaction in the irradiated samples was found (E and F). Negative controls for each receptor are also presented (G and H).

**Figure 4:**
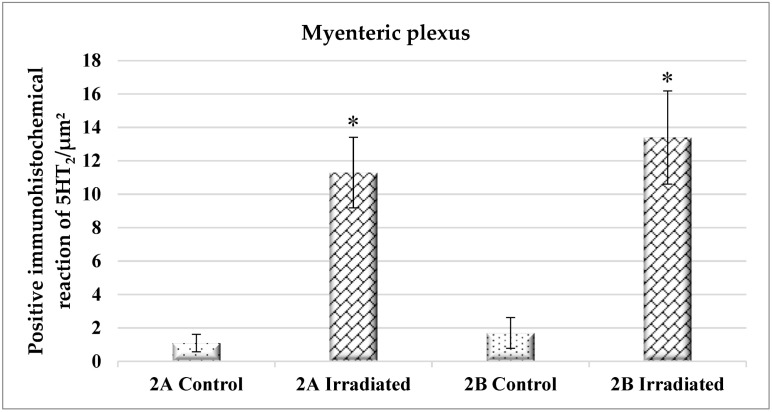
Immunohistochemical distribution of 5-HT_2A_ and 5-HT_2B_ receptors in the myenteric plexus of the gastric wall. The comparison is between 18 samples from the two groups of animals (n = 9 from each group), separately performed for each receptor type; *—*p* < 0.05.

The quantitative measurements of both receptors are presented in Figure 4. There was a significant increase of 5-HT_2A_ and 5-HT_2B_ receptors in the myenteric plexus from irradiated probes compared to the controls (Figure 4).

### 2.6. NOS-System and Electrically Induced Mechanical Reactions of Control Samples

Due to the relaxation phase of the control SM samples, an assessment of nitric oxide (NO) participation in electrically stimulated mechanical reactions was made functionally by L-arginine, a substrate of nitric oxide synthase (NOS), and its blocker L-nitro-arginine methyl ester (L-NAME). Determination of the strength and duration of contraction/relaxation phases was performed. The results are presented in Table 4.

Upon application of 5 × 10^−4^ mol/L L-arginine, the contraction phase of control SM samples did not change significantly, but there was a statistical increase in the amplitude of the relaxation phase (Table 4). The contraction phase duration was significantly reduced to 42.11 ± 5.21 s, and a tendency to relaxation phase extension (286.8 ± 63.2 s) was registered.

In the background of 5 × 10^−4^ mol/L L-NAME, the relaxation phase of the control samples was absent. There was a tendency for an increase in the duration of electrically induced contraction of 53.0 ± 7.0 s compared to the initial values after application with L-NAME.

## 3. Discussion

Our results show no significant differences between parameters of spontaneous phasic contractions of SM isolated from irradiated and control animals. The lack of difference indicates that five days after irradiation, the cellular structures responsible for these contractions—interstitial cells of Cajal [20] and contractile apparatus of SM cells—function normally. Since neuronal structures are not directly involved in the generation of spontaneous phasic contractions [21], the above results cannot categorically rule out possible neuronal damage because of irradiation. A comparison of SM responses caused by the neuronal electrostimulation of control and irradiated samples could assess the irradiation-induced changes in different neurotransmitter circuits, which impact the contractility of SM cells. These mechanical SM responses in both studied groups are of relatively equal duration and biphasic: an initial contractile peak phase, followed by relaxation in the controls and a second contraction in the irradiated group. Since these responses were prevented by TTX, a blocker of axonal conduction, they should have a neuronal genesis [22]. They result from the activation of different categories of intramural neurons, leading to the local release of neurotransmitters that can have a stimulating or suppressing effect [23]. These substances interact with various cell types in the tissues, whereby each of them, in addition to direct action on SM cells, can influence processes of production and release of other neurotransmitters by other motor neurons [24], causing a complex final SM reaction.

The contractile phase of the control samples was tendentiously suppressed by 4-DAMP and atropine. This suggests that the contraction may be provoked mainly by the electrically induced release of acetylcholine from neurons of the myenteric neurons and the interaction of the latter with muscarinic receptors (m-AChRs) on SM cells. We hypothesized an involvement of non-cholinergic excitatory neurotransmitters (like serotonin). Our assumption has been confirmed by minimizing the amplitude of the contraction phase in the presence of ketanserin (blocker of 5-HT_2_ receptors, the activation of which contracts gastric SM) [25]. Electrostimulation with the used parameters activates serotonergic neurons. In the presence of a significant percentage of such neurons in the myenteric plexus [26], the act of electrical stimulation induces serotonin release, which affects SM motility and forms a part of the contractile phase. The present relaxation phase in the control samples develops about a minute after the start of electrical stimulation. Its relatively later development is indicative of a genesis resulting from neuron–neuronal interaction. There is evidence that ENS neurons that secrete acetylcholine are excitatory in terms of the release of other mediators [27].

Given the recorded activation of cholinergic neurons after electrical stimulation, this is most likely due to the acetylcholine-induced release of NO from nitrergic neurons, an agent inhibiting contractile SM activity [28]. This hypothesis was supported by the recorded reduction of the relaxation phase after blockage with 4-DAMP of m1-AChRs and m3-AChRs, localized in myenteric nitrergic nerve cells [29] and the complete lack of such relaxation in the background of the nonselective cholinolytic antagonist atropine.

The increase in the amplitude of the electrostimulated relaxation phase registered in the background of ketanserin in control preparations can be associated with a reduction in the contractile effect. After blocking 5-HT_2_ receptors, the serotonin released from the neurons dominantly realizes its effect through other kinds of 5-HT receptors, some of which relax SM, combining with the expanded conditions of the nitrergic-induced relaxation effect discussed above.

Also, our experiments with L-arginine and L-NAME point out that NO is the leading signaling molecule of the relaxation phase. L-arginine is a pivotal substrate for NO synthesis [30]. It stimulates synthesis and increases NO levels. Therefore, the relaxation phase strength significantly increases in the presence of L-arginine with a tendency to lengthen over time. Conversely, in the background of 5 × 10^−4^ mol/L L-NAME, an antagonist of NOS [31], the duration of the electrically induced first contractile phase increased, and the relaxation phase was practically absent, similarly to the electrically induced reactions after the application of atropine.

As noted above, electrical stimulation of SM samples from irradiated animals causes a biphasic, contractile/contractile response. In terms of strength and duration, its first phase was similar to that of the control preparations. Experimental data show that the main provoking substances are identical—acetylcholine and serotonin. However, compared to the reaction in non-irradiated animals, the proportion of serotonin participation notably increases the effectiveness of ketanserin in reducing the amplitude of the contractile phase. The latter is also confirmed by our immunohistochemical studies, which show a reliable increase in the immunohistochemical reaction of 5-HT_2_ receptors in the irradiated samples (myenteric plexus) and SM cells, which are in contact with it [12] and the consequent enhanced serotonin signaling and efficiency five days after irradiation.

The participation of acetylcholine (ACh) in the development of the contractile phase recorded for irradiated SM tissue is reduced (Table 2). The effectiveness of m-AChR blockers in reducing this phase is weaker compared to their influence in controls. Radiation-increased cholinesterase activity probably contributes to this effect.

Apparently, in the irradiated tissues, the mix of mediators released by the excitatory neurons, as a result of the electrical stimulation, does not produce a relaxation phase. Our studies do not exclude the possibility of radiation-induced disturbances during NO release from nitrergic neurons. The registered second contractile response is a consequence of the increased number of 5-HT_2_ receptors and their involvement in the contractility of the SM. An argument in this meaning is its significant reduction after the preliminary blocking of 5-HT_2_ receptors. The augmenting effect of serotonergic transmission observed in the irradiated samples is likely compensatory for the relaxing effect of NO seen in the controls.

## 4. Materials and Methods

### 4.1. Research Design

To elucidate the strength and nature of radiation influence, we compared and analyzed the effects of ionizing radiation five days after irradiation on the mechanical reactions of SM tissue samples (isolated from gastric corpus) compared to control ones. We used electrical stimulation on the samples to prove the activation of part of this from ENS structures (myenteric plexus).

Application of electrical stimuli of shorter than 1 ms pulse width specifically excites neurons, followed by the release of neurotransmitter substances influencing SM contractile activity [32]. The neurogenic nature of the electrically stimulated SM responses was tested and confirmed by pretreatment of the samples with tetrodotoxin (TTX), a selective blocker of neuronal fast voltage-gated sodium channels.

We focused on changes in cholinergic, serotonergic, and non-adrenergic, non-cholinergic (NANC) systems. The role of m-AChRs in the electrically evoked mechanical responses from the two groups of tissues was clarified by using muscarinic antagonists: atropine and 1,1-dimethyl-4-diphenylacetoxypiperidinium iodide (4-DAMP). Also, we performed a comparison between the AChE activity in control and irradiated tissue homogenates.

Since electrostimulation-induced mechanical SM responses are mainly contractile and the fact that 5-HT_2A_ and 5-HT_2B_ receptors respond with SM contraction to the action of serotonin [33,34,35,36], our study aimed to assess the influence of electronic radiation on the above serotonin receptors. For this purpose, a comparative study was performed on two types of tissues: control (non-irradiated) and subjected to radiation. To evaluate the involvement of 5-HT_2A_ and 5-HT_2B_ receptors in the myenteric plexus of control and irradiated samples, we used an immunohistochemical method and electrostimulation-induced SM reactions in the background of ketanserin.

The activity of SM cells from the GI tract is controlled by neurons in the myenteric plexus, with relaxation mediated primarily by the NANC system. It is known that electrically evoked relaxations are mediated by the inhibitory neurotransmitter (neuromodulator) NO [37,38]. The presence of a relaxation phase during the electrostimulation of control samples was the basis for investigating the involvement of NO and NOS in our experiments to clarify the radiation-induced changes in the NANC-ergic system. After electrostimulation of irradiated and control samples, SM responses were investigated upon application of the NO precursor L-arginine and L-NAME (an inhibitor of its synthesizing enzyme family (NOS)).

### 4.2. Drugs and Chemicals

Serotonin (5-HT), acetylcholine, atropine, L-arginine, L-γ-nitroarginine methyl ester (L-NAME), 5,5′-dithiobis(2-nitrobenzoic acid) (DTNB), and acetylcholinethioiodide were products of Merch-Sigma-Aldrich, Darmstadt, Germany. 4-DAMP (Merch-Sigma-Aldrich, Germany), ketanserin (Merch-Sigma-Aldrich, Germany), ketamine (Richter Pharma AG, Wels, Austria), xylazine (Alfasan Diergeneesmiddelen BV, Woerden, The Netherlands), and tetrodotoxin (TTX) (Sankyo, Zurich, Switzerland) were also used. Mouse monoclonal antibodies SR-2A (A-4): sc-166775. or SR-2B (C-6): sc-376878 (Santa Cruz Biot., Inc., Santa Cruz, CA, USA), buffer for rinse (50 mM TRIS, pH 7.6, 150 mM NaCl_2_, 0.05% Tween-20, TTBS), the Biotin Blocking Kit (cat. N° BBK 120, ScyTek Laboratories, Inc., Logan, UT, USA) 3,3′-diaminobenzidine tetrahydrochloride (DAB, cat. N° ACV500 Scy Tek., USA), alcohol solutions (70%, 80%, 96%, 100%), and xylene were obtained from the specified sources. The Krebs solution (pH 7.4) was composed of 120 mM NaCl, 5.9 mM KCl, 2.5 mM CaCl_2_, 1.2 mM MgCl_2_, 1.2 mM NaH_2_PO_4_, 15.4 mM NaHCO_3_, and 11.5 mM glucose.

### 4.3. Animals and Anesthetic Protocol

The animal experiments were carried out according to European Union (Directive 2010/63/EU) and Bulgarian guidelines (Directive No. 20/01.11.2012) for using laboratory animals (License No. 213/5.10.2018 from the Animal Health and Welfare Directorate of the Bulgarian Food Safety Agency (BFSA, https://bfsa.egov.bg/wps/portal/bfsa-web-en/home (accessed on 16 May 2024).

Male Wistar rats (n = 16) with body weights between 250 and 280 g were provided by the Animal House of Medical University—Plovdiv, Bulgaria. The rats were housed under standard laboratory conditions (23–25 °C, 50–55% humidity, 12/12 h light/dark cycle), fed with standard commercial food and given water ad libitum. At the beginning of the irradiation procedure, they were anesthetized using 2% xylazine (10 mg/kg) + 5% ketamine (calipsol) (100 mg/kg), administered as an intraperitoneal injection.

### 4.4. Irradiation Procedures

The irradiation procedure was performed as described in our previous study [39]. Briefly, anatomical topographic planning involved selecting an optimal rat position for consistent and reproducible irradiations. Treatment planning was performed using a Siemens Somatom Spirit Power CT scanner and CMS XiO system for three-dimensional dosimetry. Rats were anesthetized and immobilized in a dorsal position on a plexiglass mat (30 × 30 × 0.5 cm).

A Siemens Primus S/N 3561 linear accelerator was used for total body irradiation with a 9 MeV electron beam. A standard 25 × 25 cm symmetrical electron applicator delivered a single dose of 5 Gy at a depth of 2 cm. The procedures were overseen by a medical physicist, radiotherapist, and radiographers.

Rats (n = 8) were centered abdominally before irradiation to ensure consistent positioning. They were monitored visually throughout the session and transported to the laboratory for further investigations afterward.

### 4.5. Isometrical Registration of SM Contractile Activity

The SM contractile activity was registered isometrically and as previously described [39]. In brief, the animals were euthanized by overdose anesthesia (five times greater doses of ketamine and xylazine than used for anesthesia—see Section 4.3) and SM strips from the circular muscle layer of the stomach corpus were cut (width of 1.0–1.1 mm; length of 13–15 mm).

For the isometrical registration, we used tenso detectors (“Swema”, Sweden). The initial mechanical tension of the preparations was achieved and corresponded to a tensile force of 10 mN. Krebs solution, used for bathing SM preparations, was continuously aerated with a gas mixture of 95% O_2_ and 5% CO_2_ at 37 °C. Changes in muscle tones (contraction or relaxation) were compared to the tone level, obtained after initial 60 min adaptation. During the period of adaptation, Krebs solution was changed several times. Drug-evoked reactivity of SM preparations was registered by gain stage (“Microtechna”, Czech Republic) and recorded on paper recorder (“Linseis”, Germany).

### 4.6. Electrical Stimulation

The electrostimulation of all samples was carried out by rectangular pulses with the following parameters: duration of the individual pulse 200 μs, frequency 20 Hz, duration of stimulation 50 s, and amplitude of the signal at the output of the electrostimulator 60 V. These parameters were based on preliminary studies in our laboratory that revealed their potential to cause mechanical SM responses with neurogenic genesis.

During each experiment, successive electrical stimulations of SM samples were made at intervals of 10–15 min necessary to restore spontaneous contractile activity [40].

### 4.7. Determination of Acetylcholinesterase (AChE) Activity

AChE activity was measured according to the optimized Ellman method as described elsewhere [41,42]. Briefly, thiocholine released after enzymatic hydrolysis of the substrate acetyl thiocholine reacts with 5,5′-dithiobis(2-nitrobenzoic acid) (DTNB) and forms colored product absorbing at 412 nm. The amount of this product is proportional to AChE activity. The enzymatic reaction was started by adding 0.1 mL tissue homogenate to 0.8 mL DTNB dissolved in sodium phosphate buffer and 0.1 mL substrate. The increase in the absorption at 412 nm was followed over 3 min.

One unit of activity is defined as the amount of enzyme hydrolyzing 1 μmol of acetylthiocholine iodide per minute at 25 °C and pH 7.0. The activity is calculated using the formula:

U=ΔAmin13.88,

where 13.88 is the molar absorptivity (mM^−1^ × cm^−1^) of the color product formed. The specific activity of AChE represents the activity of the enzyme related to the amount of protein (mg) in 0.1 mL tissue homogenate:

U′=Umg of protein

### 4.8. Immunohistochemical Methods

The immunohistochemical studies were performed on paraffin-embedded sections (thickness—5 µm) obtained from SM tissues of control and irradiated rats (n = 6), mounted on glass microscope slides, and secured using an adhesive.

The sections were prepared from isolating fragments (5 × 5 mm) of the gastric wall, followed by fixing in 10% neutral buffered formalin for 48 h. After dehydration in alcohol of an ascending concentration (70%, 80%, 96%, 2 × 100%) and clarification in cedarwood oil, they were embedded in paraffin at 56 °C for 12–18 h.

Following deparaffinization and rehydration, the sections were washed 3 times for 5 min each in a wash buffer (50 mM TRIS, pH 7.6, 150 mM NaCl_2_, 0.05% Tween-20, TTBS) and incubated for 10 min with 3% dH_2_O_2_. An appropriate kit (catalog N° BBK 120, ScyTek, USA) was used to block endogenous biotin; the sections were incubated according to the kit protocol: 15 min with part A, then washed, and then 15 min with part B. This was followed by washing with buffer three times for 5 min, after which the sections were incubated at room temperature for 5 min with a reactive to block the non-specific binding (Superblock), were given a single wash, and were incubated for 24 h at 4 °C with a specific mouse monoclonal antibody against SR-2A (A-4) or SR-2B (C-6), (Santa Cruz, CA, USA, 1:150 solution). The procedures that followed included: washing with TTBS (3 × 5 min); incubation with a biotinylated anti-mouse secondary antibody at room temperature and a second washing with TTBS (3 times for 5 min). The reaction was visualized using 3,3′-diaminobenzidine tetrahydrochloride (DAB) (ScyTek Lab. Inc., USA) in the dark (2 × 5 min) and washing the sections in distilled water twice for 5 min. After that, the preparations were counterstained with hematoxylin, differentiated in running tap water, dehydrated in alcohols of an ascending concentration, incubated in xylol (3 times for 5 min), and then covered with Canada balsam. Negative controls for both receptors were run in parallel by omitting the primary antibody under the same conditions.

The preparations were observed under a light microscope, magnification ×200.

### 4.9. Quantitative Analysis of the Immunohistochemical Reactions for 5-HT_2A_ and 5-HT_2B_

The positive immunohistochemical reactions in the gastric wall smooth muscle cells were recorded within the image parameters previously determined. Each cell that was immunohistochemically positive for the 5-HT_2A_ and 5-HT_2B_ receptor was marked as counted using the specialized software Olympus DP-Soft Image System (version 4.1 for MS Windows) of a Microphot-SA microscope (Nikon, Tokyo, Japan) provided with a Camedia-5050Z digital camera (Olympus, Tokyo, Japan). The analysis was performed on gastric sections of Wistar rats (n = 6) for both groups of animals: control and irradiated. Analysis of each antibody was made involving 5 fields (sized 100 µm^2^) of the gastric muscle layer (tunica muscularis) of each animal; the average number of cells with a positive reaction per square unit was determined in each field using a measuring mesh (6 × 5 fields), at a magnification ×200 [43].

### 4.10. Statistical Analysis

The number of tissue preparations used in each experiment is indicated by n. At the start of statistical analysis, the Shapiro–Wilk test was performed for normal distribution. Statistical differences were tested using the *t*-test, paired *t*-test, and Welch test when n is different for all groups. The data obtained are expressed as mean ± standard deviation. A probability of *p* < 0.05 was considered significant. All statistical analyses were performed using SPSS software, version 17.0 (SPSS Inc., Chicago, IL, USA).

## 5. Conclusions

Our research demonstrates that the 5 Gy absorbed dose of accelerated electron radiation affects neuronal signaling in the myenteric plexus 5 days post-irradiation procedure. An expression of this is a change in the nature of electrostimulation-induced SM responses due to a change in the ratio between substances released by electrostimulating neurons. The results point toward a reduced contribution of the cholinergic system to contractile processes in irradiated tissues versus control and a lack of the ACh-induced NO relaxation phase characteristic of control samples. Electron radiation activates the serotonergic neurotransmitter system, which is expressed in an increase in its share in electrostimulation-induced processes.

## Figures and Tables

**Table 1:** Comparative characteristics of spontaneous phasic contractions of SM samples from control and irradiated rats. Data are presented as mean ± standard deviation.

Parameters of Spontaneous Contractile Activity	SM Probes	
	Controls (n = 15)	Irradiated (n = 9)
frequency, min^−1^	5.02 ± 0.19	4.93 ± 0.26
amplitude, mN	1.88 ± 1.01	1.62 ± 0.96

**Table 2:** The amplitude of biphasic electrical-evoked responses of control and irradiated samples; initial values after electrostimulation and values after treatment with 4-DAMP and atropine. The “–” sign in front of the numerical values indicates a relaxation process and n indicates the number of samples.

Experimental Animals	Nature of SM Reaction	Electrically Induced SM Reactions, mN					
		Initial Values	n	After 4-DAMP (2 × 10^−6^ M)	n	After Atropine (1 × 10^−6^ M)	n
Controls	I phase—contraction	19.3 ± 3.23	9	5.44 ± 1.37	5	6.05 ± 1.98	5
	II phase—relaxation	−1.87 ± 0.64		−0.43 ± 0.37		0	
Irradiated	I phase—contraction	21.90 ± 7.20	7	10.50 ± 3.2 *	5	11.34 ± 3.09	6
	II phase—contraction	1.35 ± 0.33 *		2.45 ± 1.36		2.66 ± 0.72	

Note: (*) indicate statistically significant differences (*p* < 0.05). (*) refers to the comparison between values measured for control and irradiated samples at one and the same experimental conditions.

**Table 3:** The amplitude of biphasic electrical-evoked responses of control and irradiated samples after administration of ketanserin. The “–” sign in front of the numerical values indicates a relaxation process and n indicates the number of samples.

Experimental Animals	Nature of SM Reaction	Electrically Induced SM Reactions, mN	
		Initial Values	After Ketanserin
Controls n = 9	I phase—contraction	19.3 ± 3.23	14.5 ± 6.4
	II phase—relaxation	−1.87 ± 0.64	**−2.8 ± 0.35 ^^^**
Irradiated n = 7	I phase—contraction	21.90 ± 7.20	9.50 ± 3.2 ^^,^*
	II phase—contraction	1.35 ± 0.33 *	**0.59 ± 0.7 ^^^**

Note: (^) and (*) indicate statistically significant differences (*p* < 0.05). (^) refers to the comparison between the initial value of each of the phases for control and for irradiated samples and their corresponding values after the application of blockers; (*) refers to the comparison between values measured for control and irradiated samples at one and the same experimental conditions.

**Table 4:** Comparison of the amplitudes of two-phasic electrically elicited SM responses of tissues dissected from control animals after initial electrical muscle stimulation and in the presence of L-arginine and L-NAME. The “–” sign in front of the numerical values indicates a relaxation process and n indicates the number of samples.

Nature of SM Reaction	Electrically Induced SM Reactions, mN					
	Initial Values	n	After L-Arginine (5 × 10^−4^ M)	n	After L-NAME (5 × 10^−4^ M)	n
I phase—contraction	19.3 ± 3.23	9	17.21 ± 1.95	7	**22.68 ± 1.98 ^**	7
II phase—relaxation	−1.87 ± 0.64		**−2.61 ± 0.79 ^**		**0 ^**	

Note: (^) indicate statistically significant differences (*p* < 0.05). (^) refers to the comparison between the initial value of each of the phases and their corresponding values after the application of blockers.

## Data Availability

All materials are presented in the manuscript.

## Supplementary Materials

The supporting information can be downloaded at: https://www.mdpi.com/article/10.3390/ijms25126807/s1.
