# Supplementary material for: Accelerated Electron Ionization-Induced Changes in the Myenteric Plexus of the Rat Stomach
Source: Int J Mol Sci. 2024 Jun 20;25(12):6807. doi: 10.3390/ijms25126807 (PMC11203758; doi:10.3390/ijms25126807)
Supplement: Supplementary file 1 [file ijms-25-06807-s001.zip › ijms-3039762-supplementary.pdf]

„Образец ЗХОЖ – 47/ Утвърден със Заповед № РД 11-2211/14.11.2016 г. на изпълнителния директор на БАБХ

РЕПУБЛИКА БЪЛГАРИЯ
МИНИСТЕРСТВО НА ЗЕМЕДЕЛИЕТО, ХРАНИТЕ И ГОРИТЕ
БЪЛГАРСКА АГЕНЦИЯ ПО БЕЗОПАСНОСТ НА ХРАНИТЕ

РАЗРЕШИТЕЛНО
за използване на животни в опити № 213

Настоящото разрешително се издава на **проф. д-р Стефан Костянев, дм, дмн, Ректор на МУ – Пловдив** (име, презиме и фамилия на собственика/управителя на обекта, в който се провеждат опити с животни)

на обекта с вет. регистрационен № **4000-0157**

и адрес: гр. Пловдив 4002, бул.''Васил Априлов'' № 15 А
(седалище, адрес на управление на юридическото лице)

БУЛСТАТ 000455471

за използване на **170 (сто и седемдесет) броя плъхове, порода Wistar.**
(вид и брой опитни животни)

Целта на опитите/научните изследвания, за които ще се използват животните е за **''Радиационни ефекти върху биоелектрични и механични процеси в гладкомускулни клетки и тъкани на гастроинтестинален тракт на целотелно облъчени с електронен сноп (линеен ускорител siemens primus He 3561) плъхове.''**

Ръководителят на екипа, който ще провежда опитите/научните изследвания е: **проф. Атанас Кръстев; проф. Жанет Грудева-Попова, дмн**

За спазването на изискванията за хуманно отношение към опитните животни ще отговарят:

д-р Радка Абрашева-Тодорова

Разрешението е вписано в регистъра на БАБХ за използване на опитни животни с **регистрационен № 213 и е валидно до 11.10.2023 г.**

Настоящото разрешително се издава на основание чл. 155, ал. 7 от ЗВД и становището на комисията по етика № 129 от 05.10.2018 г., неразделна част от разрешението.

Д-Р ДАМЯН ИЛИЕВ
ИЗПЪЛНИТЕЛЕН ДИРЕКТОР

БЪЛГАРСКА АГЕНЦИЯ ПО БЕЗОПАСНОСТ НА ХРАНИТЕ
Република България
